# Low knowledge levels and high willingness to use oral Pre-Exposure Prophylaxis (PrEP) among Key Populations in Kampala, Uganda: Implications for targeted educational interventions

**DOI:** 10.21203/rs.3.rs-4943952/v1

**Published:** 2024-09-26

**Authors:** Bashir Ssuna, Mari Armstrong-Hough, Maiya G Block Ngaybe, Dennis Kalibbala, Joan N Kalyango, Flavia Matovu Kiweewa

**Affiliations:** Makerere College of Health Sciences, Department of Epidemiology and Biostatistics, Kampala, Uganda; Uganda Tuberculosis Implementation Research Collaboration (U-TIRC), Kampala, Uganda; Uganda Tuberculosis Implementation Research Collaboration (U-TIRC), Kampala, Uganda; New York University School of Global Public Health, Department of Social and Behavioral Sciences, Department of Epidemiology, New York, United States; University of Arizona, Mel and Enid Zuckerman College of Public Health, Health Promotion Sciences Department, Arizona, United States; Makerere College of Health Sciences, Department of Epidemiology and Biostatistics, Kampala, Uganda; Global Health Uganda, Kampala Uganda; Makerere College of Health Sciences, Department of Epidemiology and Biostatistics, Kampala, Uganda; Makerere College of Health Sciences, Department of Epidemiology and Biostatistics, Kampala, Uganda; Makerere University-John Hopkins University Research Collaboration (MU-JHU), Kampala, Uganda

**Keywords:** Preexposure prophylaxis, fisherfolk, MSM, female sex workers, health knowledge, key populations, Sub Saharan Africa

## Abstract

**Background:**

Preexposure prophylaxis (PrEP) reduces new human immunodeficiency virus(HIV) infections by up to 96% and is recommended for key populations by the World Health Organization. Understanding the knowledge and willingness to use PrEP is essential for effective implementation. This study assessed these factors and identifiedcharacteristics associated with differences in knowledge among key populations in Kampala, Uganda.

**Methods:**

We administered a cross-sectional survey to a systematic sample of 497 participants from fisherfolk (283, 56.9%), men who have sex with men (MSM) (93, 18.7%), and female sex worker (FSW) (121, 24.4%) communities in Kampala Central, where PrEP had not yet been rolled out. Data on sociodemographic characteristics, PrEP awareness, and HIV-related behavioralfactors were collected. Knowledge about PrEP was measured using an adopted questionnaire comprising five key questions about PrEP knowledge, graded as no knowledge, some knowledge and good knowledge. Ordered probit regression models were used to analyze the associations of independent factors with PrEP knowledge levels.

**Results:**

Participants had a mean age of 29±7.6 years. Ofthese, 257 (51.7%) reported having sex with women, 157 (31.6%) with men and 83 (16.7%) with both men and women. Self-reported HIV-positive status was 6.4% in fisherfolk, 11.8% in MSM and 27.3% in FSW. PrEP awareness stood at 62.4% overall, with the highest awareness in FSW (73.6%) and the lowest in fisherfolk (54.1%). Willingness to use PrEP was high across all groups (77.7%), although it was lower among FSW (66.9%). Multivariate probit analysis highlighted key independent factors associated with PrEP knowledge among fisherfolks and HIV-related concerns (Adj. Coeff = 0.54, 95% CI: 0.11, 0.97) and lack of PrEP awareness (Adj. Coeff = −0.99, 95% CI:−1.28, −0.70); among MSM, lack of PrEP awareness (Adj. Coeff = −1.74, 95% CI:−2.38, −1.10); and in FSW, tertiary education (Adj. Coeff = 1.53, 95% CI: 0.07, 2.99) and marital status (Adj. Coeff = 0.57, 95% CI=0.09, 1.05). MEshighlighted the influence of these independent factors at each knowledge level.

**Conclusions and Recommendations:**

Despite the high willingness to use PrEP, significant knowledge gaps exist, particularly concerning dosage and use duration, which are influenced by factors such as HIV concern and educational attainment. Tailored educational initiatives may bridge these gaps and enhance willingness to use PrEP.

## Introduction

Globally, key populations continue to bear a disproportionate burden of HIV infection. Despite being less than 5% of the global population, key populations and their sexual partners account for 55% of the global population with new HIV infections in 2022[1, 2]. According to UNAIDS data from 2022, the median HIV prevalence among these groups significantly exceeded that of the general adult population (aged 15–49), which was 0.7%. In contrast, the prevalence of HIV was 2.5% among female sex workers, 7.5% among men who have sex with men, and 10.3% among transgender people[3]. Uganda still has a relatively high HIV prevalence rate—5.1% among adults—compared to the worldwide average of 0.6% [4, 5]. Kampala is the capital and largest city of Uganda, and the city has five divisions, i.e., the Kampala Central Division, Kawempe Division, and Makindye, Nakawa and Lubaga Divisions. The total population of Kampala is 1.6 million people[6]. The prevalence of HIV in Kampala has most recently been recorded to be even higher than the national average, at 6.9% in 2016[7] and 6.0% in 2023[8]. Recent data from Uganda from 2014–2016 revealed even greater percentages of fishing communities (10.8%−28.8%)[9–11], female sex workers (44%)[12], and MSM (18%)[13].

The World Health Organization (WHO) advocates for the use of PrEP[14], which has shown efficacy rates up to 99% in reducing HIV transmission among high-risk groups[15–17]. In 2017, PrEP started to spread to key populations in Uganda[18, 19]. Despite its proven efficacy, uptake, and willingness to use, PrEP varies significantly globally and is influenced by multiple factors, including knowledge levels, perceived HIV risk and sociocultural attitudes[20, 21]. Poor knowledge about PrEP and negative perceptions are associated with low acceptability of PrEP in these HIV key populations, which influences its adherence and effectiveness[21, 22].

Recent studies in Uganda among key populations have reported low levels of awareness of PrEP (35%−48.2%) [20, 23], low levels of HIV testing and status awareness (54%), and low levels of condom use (4%), despite having a higher HIV incidence and greater HIV risk transmission [24]. Determining potential users’ knowledge, willingness to use and associated factors and knowledge levels of PrEP is a critical step in its successful implementation because it will enable the design of targeted outreach. This study aimed to fill this gap by exploring the differences in the knowledge about PrEP and willingness to use it across three different key population groups in Kampala: fisherfolk, MSM, and FSW. We utilized a cross-sectional survey to examine how demographic factors, HIV-related behaviors, and PrEP awareness shape PrEP knowledge levels.

## Materials and Methods

### Design and setting

We carried out a cross-sectional survey of key populations in Kampala District, Uganda, from 5th February to 4th June 2019. Kampala is the capital and largest city in Uganda; the prevalence of HIV was 6.9% in Kampala in 2016[7]. This study was conducted in the Nakawa, Rubaga and Makindye divisions of Kampala. Female sex workers are mostly found on bars and on streets in the city and in most at-risk population (MARP) clinics. The Ggaba landing site was used as the fishing community in Kampala in this study and had a population of approximately 17,000 people in 2016[25]. The fisherfolk population is highly transient and has a high HIV transmission rate of 3.39 per 100 person-years at risk compared to 0.46 per 100 person-years in the general population[26–28]. The MARPs clinic of Mulago Hospital is supported by the Infectious Disease Institute (IDI), Baylor Uganda and the Uganda Ministry of Health. The clinic receives more than 100 FSWs and 90 MSMs within three months.

### Study population, sample size and sampling

We included adults aged 18 and above who were identified as MSM or FSW and who were residents at the Ggaba landing site; who lived in that location for at least three months; and who provided informed consent to participate in the study. We excluded individuals who were too ill to participate in the study.

The sample size for each population was determined using a comparison of two proportions (Fleiss formula)[29]. We set the power at 80% (Z_β_ =0.84) and the alpha at 0.05 (Z_α_ =1.96). Additionally, based on previous studies by Jayakumaran et al., 2016 and Frankis et al., 2016[30, 31], we estimated a minimum sample size of 283 participants for fisherfolk, 93 for MSM, and 121 for FSW. We used a systematic sampling method for the survey of fisherfolk and snowball sampling for MSM and FSWs because of the importance of confidentiality due to existing persecution laws against these populations in the country. We estimated a population of 1000 adults at the Ggaba landing site and generated a sampling fraction of four with a random number between one and four to generate a sampling interval of three. We included every fourth adult in the survey, beginning with the third adult, until the sample size was reached.

### Data collection tools and methods

Trained interviewers administered a semistructured questionnaire with both open- and closed-ended questions using the Open Data Toolkit (ODK) in English and/or Luganda, the local language. The questionnaire was electronic, and interview questions were collected using a tablet. The knowledge questionnaire included five simple questions about the use of PrEP as an HIV preventive measure, on PrEP dosing, and on the effectiveness of PrEP. The questionnaire about willingness to use PrEP and independent factors was adopted from the WHO HIV risk indicators and previous literature about the acceptability of PrEP. All the questionnaires used in the study were pretested on 15 random participants from each key population who were not included in the study before administration among the study participants. Willingness to use PrEP when provided was measured using “yes” or “no”. Awareness of PrEP being rolled out was measured as “yes” or “no”.

### Statistical analysis

We produced descriptive statistics such as proportions and means ± standard deviations for all the variables. The primary outcome was knowledge about using oral PrEP, which was measured as both continuous (0–100%) and categorical (0–20%), as was knowledge (40%−60%) and good knowledge (≥ 80%). Willingness to use PrEP and awareness were measured as a proportion with 95% confidence intervals. Because the populations were different, bivariate and multivariate analyses were performed for the specific key populations. An ordered probit regression model was used at both levels to determine the coefficients and 95% confidence intervals (CIs) for factors influencing knowledge about PrEP. Factors with a P value < 0.2 at the bivariate level were further assessed at the multivariate level. Confounding was determined by a difference of ≥ 10% between the crude and adjusted models. Margin estimates or effects of the probit model were also determined to assess how the respective independent factors influenced the probability of having no knowledge, some knowledge or good knowledge about PrEP. Analyses were performed using Stata Version 15.1/MP.

## Results

### Description of the study participants

A total of 497 participants were recruited for this study—283 (56.9%) from fishing communities, 93 (18.7%) from MSM communities and 121 (24.4%) from the communities of FSWs. The mean age was 29 ± 7.6 years. Of these, 257 (51.7%) reported having sex with women, 157 (31.6%) with men and 83 (16.7%) with both men and women. Participants from the key population communities differed in age [P < 0.001], location [P < 0.001] and level of education [P < 0.001]. Participants also differed with respect to HIV testing in the past 6 months (P = 0.001), HIV status (P < 0.001), condom use (P = 0.001), HIV risk perception (P < 0.001) and HIV concern (P < 0.001). Self-reported HIV-positive status was 18 (6.4%) for fisherfolk, 11 (11.8%) for MSM and 33 (27.3%) for FSWs; additionally, 23.5% of all the participants were unaware of their HIV status (Table 1).

### Awareness and willingness to use PrEP

Awareness of PrEP being used as an HIV preventive measure in their respective communities was 62.4% for all the key populations. Awareness was highest among FSWs (73.6%), followed by MSM (73.1%), and only 54.1% of the fisherfolks were aware of PrEP. However, the willingness to use PrEP among all the key populations was high, with an overall willingness of 77.7% [95% CI 73.8–81.1]. There was a lower percentage of female sex workers than in the remaining key population (66.9%) [95% CI 57.8–75.2].

### Knowledge about PrEP as an HIV preventive method

**Figure 1:**
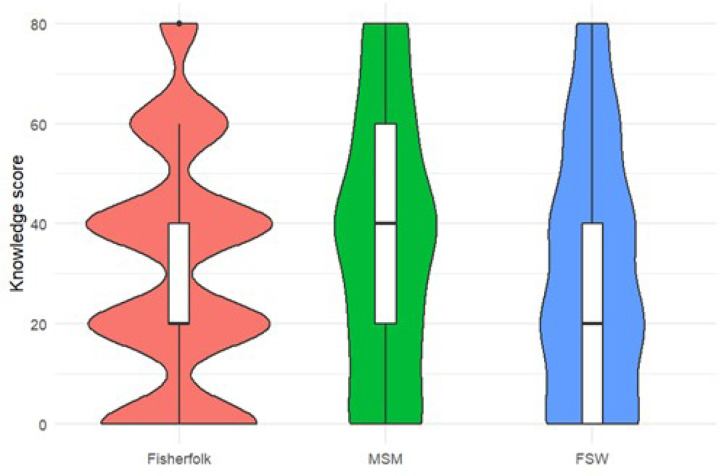
Violin plots showing the distribution of median and interquartile rages for the knowledge scores from the key populations

**Figure 2:**
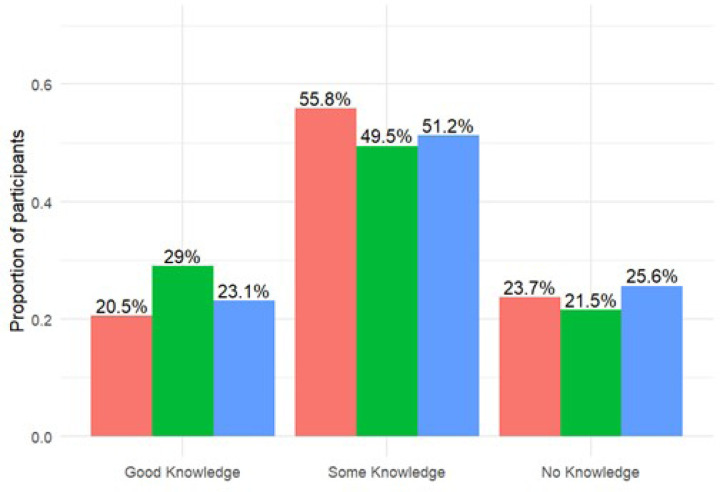
A bar graph showing proportion of participants according to knowledge categories

The mean and standard deviation of the continuous knowledge scores were 31.3 ± 24.4. Among the participants, 266 (53.5%) had some knowledge, 113 (22.7%) had good knowledge, and 118 (23.7%) had no knowledge of PrEP. MSM generally had better knowledge about PrEP than did the other key populations (Fig. 1 and Fig. 2).

According to the PrEP knowledge assessment questions, the most common uncertainties concerned the correct dose of PrEP, as 59.8% of the respondents were unsure about the duration of PrEP use, whereas 93.4% of the respondents were unclear about how long PrEP should be used. Additionally, 62.0% were unsure about whether PrEP completely prevents HIV infection. (Table 2). The reliability coefficient was 0.61 for fishing communities, 0.71 for FSWs and 0.67 for MSM. The most common source of PrEP information reported was friends (108, 21.7%), followed by health workers (105, 21.1%) and the internet (29, 5.8%).

### Knowledge and willingness to use PrEP

Having good knowledge about PrEP was associated with a 5-fold increase in willingness to use PrEP (crude OR = 5.26 [95% CI = 1.30–21.32, P = 0.020] among the FSW; having some knowledge had a 3.6-fold increase among MSM (crude OR = 3.60; 95% CI = 1.02, 12.62). Good knowledge was associated with a 2.7-fold increase in expression in fisherfolk (crude OR: 2.72, 95% CI: 0.99–7.50); however, this difference was not statistically significant.

### Associations between PrEP knowledge and independent factors

Location, education level, HIV status awareness, condom use, HIV risk perception, concerns about HIV and PrEP awareness were significantly associated with knowledge among fisherfolks, while employment, HIV risk perception, concerns about HIV and PrEP awareness were significantly associated with knowledge among MSM. Among FSWs, education, marital status, having tested for HIV in the past 6 months, concerns about HIV, and PrEP awareness were significantly associated with knowledge (Table 3).

Among fishermen, having HIV (adj coefficient: 0.54, 95% CI: 0.11,0.97) and lacking PrEP awareness (adj coefficient: 0.99, 95% CI: −1.28, −0.70) were significantly associated with knowledge about PrEP. Among the MSM population, lack of PrEP awareness (adj coefficient: −1.74, 95% CI: −2.38, −1.10), P < 0.001, was the only factor significantly associated with knowledge about PrEP.

Among the FSWs, tertiary education (adj. coefficient: 1.53, 95% CI: 0.07, 2.99), marital status (adj. coefficient: 0.57, 95% CI: 0.09, 1.05), being sometimes concerned about HIV (adj. coefficient: 1.41, 95% CI: 0.61, 2.21), and lack of PrEP awareness (adj. coefficient: −2.21, 95% CI: −2.85, −1.57) were significantly associated with knowledge about PrEP (Table 4).

### Marginal effects of factors associated with knowledge levels in key populations

The results from the ordered probit regression model show the marginal effects of various factors on different levels of knowledge within key populations. The coefficients describe how likely individuals are to have no knowledge, some knowledge, or good knowledge about PrEP based on different characteristics (Table 5). Among fisherfolks, being concerned about HIV was found to be associated with a 16% lower probability of having no knowledge about PrEP and a 12% greater probability of having good knowledge. A lack of PrEP awareness was found to be associated with a 28% greater probability of having no knowledge of PrEP and a 24% lower probability of having good knowledge. Among MSM, having a perception of HIV risk is associated with a 15% greater probability of having good knowledge about PrEP. A lack of PrEP awareness was also found to be associated with a 52% greater probability of having no knowledge about PrEP and a 36% lower probability of having good knowledge. Among the FSWs, having a tertiary education was found to be associated with a 16% lower probability of having some knowledge about PrEP and a 38% greater probability of having good knowledge. Being married or in a casual relationship was found to be associated with both a 9% lower probability of having no knowledge about PrEP and a 13% greater probability of having good knowledge. Finally, being concerned about HIV was found to be associated with a 22% lower probability of having no knowledge about PrEP and a 32% greater probability of having good knowledge.

## Discussion

In the present study, there was a general lack of knowledge about PrEP, with 23.7%−53.2% of the participants having no knowledge of PrEP, i.e., answering three or fewer of the five questions in the survey correctly. Only 22.7% of participants had good knowledge about PrEP (scored 80%). The Falk population had lower scores than did the other key populations. Poor knowledge about PrEP among these key population communities may be partly because the most common source of PrEP information was from friends (21.7%). Other explanations may include negative perceptions about PrEP and social stigma, which have been demonstrated in previous studies[32, 33]. Similar results have been reported among MSM in Denver, Colorado, where only 21% of participants were aware of PrEP[34]; this percentage was comparable to the 20% found in this study, and 15.3% was reported in Philadelphia[22]. A meta-analysis of 13 studies from low- and middle-income countries (LMICs) revealed that awareness was low (29.7%) among MSM[35], which was slightly greater than what we found in the present study. Among FSWs in China, 15.1% were aware[36], while in Thailand, this figure was as low as 10%[37], which was slightly lower than what we found in the present study. It is important to note, however, that the other studies on PrEP knowledge mentioned above assessed knowledge as “awareness”, which is not the same as how this study assessed knowledge.

In this study, 77.7% of the participants were willing to use PrEP across all the key population communities (95% CI 73.8–81.1). Almost 8 out of the 10 participants were willing to use PrEP if it was provided to them. This high willingness to use PrEP can be explained by the participants’ perceptions of high HIV risk expressed in the quantitative analysis (81.3%) and their concerns about contracting HIV from their partners.

In this study, participants who identified as MSM had the highest willingness among the three groups (82.8%, 95% CI: 73.6, 89.3), and female sex workers had the lowest willingness (66.9%, 95% CI: 57.8, 75.2%). The acceptability of the IPM in Peruvian populations reached 82.5%[21], which was comparable to the 82.8% observed in this study. A multicenter study in the clinics of Kenya, Uganda, Peru, India, Botswana, Ukraine and South Africa found that 61% of FSWs were willing to take PrEP[38], which was comparable to the 66.9% reported in the present study but lower than the 80% reported in Thailand[37] and 85.9% in China[36]. This difference could be due to differences in the environment and perceptions of PrEP. The slightly lower willingness to use PrEP among FSW participants may be explained by their perceived shortcomings in using PrEP over condoms, as PrEP does not protect them against sexually transmitted infections or pregnancy[39]. The other explanation may be the fear of side effects or stigma reported by FSWs who use PrEP[21, 40].

A lack of PrEP awareness was associated with lower knowledge levels across all key populations in this study. Among fisherfolk and FSWs, a lack of awareness of PrEP substantially decreased their knowledge and was associated with a 52% chance of having low knowledge about PrEP among MSM. This is because without basic awareness, individuals are unlikely to seek or retain information about PrEP and the first step toward knowledge acquisition and subsequent behavioral change[41]. Global studies have demonstrated that increasing awareness will improve PrEP knowledge, reduce negative perceptions and increase willingness to use PrEP among key populations[42–44]. This finding underscores the need for effective awareness campaigns to increase PrEP knowledge among fisherfolks, supported by the literature showing the critical role of awareness in health education.

Education level was also associated with knowledge about PrEP among the study participants. Having tertiary education is associated with a 38% greater probability of having good knowledge about PrEP among FSWs. This can be attributed to their enhanced ability to access and understand health information, which is often disseminated through formal education channels. These findings align with findings from a meta-analysis that indicated a positive correlation between education level and PrEP knowledge and willingness to use it among MSM in LMICs[45]. Studies in China and Thailand corroborate these findings, showing that FSWs with higher education levels are more knowledgeable about PrEP and more likely to use it[36, 37].

The perception of HIV risk and concerns about contracting HIV were associated with knowledge about PrEP among key populations. In this study, 15% of the MSM who perceived themselves to be at high risk of HIV infection were more likely to have good knowledge about PrEP. However, concerns about HIV were associated with 12% and 32% greater probabilities of having good knowledge among the fisherfolk and FSW, respectively. This perception drives individuals to seek out information and protective measures to mitigate their risk. Similarly, MSM who perceived a higher risk of HIV had better knowledge about PrEP. Awareness of personal risk prompts proactive health behaviors, including seeking out information on preventive measures. This finding is supported by studies showing that perceived risk is a significant predictor of health-seeking behavior among high-risk populations in various contexts[42, 44, 46]. The concern about HIV drives FSW to learn more about preventive measures, as seen in studies where high-risk perception among FSW is linked to better knowledge and use of PrEP[45].

For participants who identified as MSM, however, HIV testing was not significantly associated with knowledge. This could be because our MSM participants accessed their PrEP information through other channels beyond testing sites. However, other studies have shown a strong correlation between regular HIV testing and higher PrEP awareness and use among MSM[30]. Among FSW participants, recent HIV testing was significantly associated with increased knowledge and willingness to use PrEP. This association highlights the importance of combining HIV testing with educational initiatives to improve PrEP knowledge and uptake, as observed in studies of FSW in China[41].

Our study has several limitations. First, we relied on self-reports of HIV status, which means that we may have included HIV-positive individuals since some had not been tested in the last 6 months and could have had an infection at that time. Additionally, due to HIV stigma, some participants may have chosen not to disclose their HIV status. Another limitation was that there is no standard tool for determining knowledge levels about PrEP among key population communities, and the investigators developed their own tool by adopting it from the WHO HIV knowledge assessment tool. The tool adopted had low reliability coefficients among the MSM and fishing community participants; however, it provided a picture of the general knowledge about PrEP in these communities.

Our study has several strengths. First, the study participants from fisherfolk communities were systematically sampled from fishing sites to ensure high representativeness of the community. The study additionally reports crucial information about populations who are notoriously difficult to sample and who hold the key to the HIV pandemic and may help guide future interventions aimed at increasing PrEP knowledge and future uptake. The knowledge assessment tool we adopted can be used as a step in the direction of PrEP knowledge assessment instead of measuring it as awareness.

## Conclusions and Recommendations

Despite the high willingness to use PrEP across all key population communities, the knowledge about PrEP was heterogeneous. Many participants were unfamiliar with the dosing of PrEP, the duration of PrEP use, and whether PrEP completely prevents HIV infection.

A comparative analysis of factors associated with knowledge and willingness to use PrEP among fisherfolks, MSM, and FSW identified several factors: education level, HIV risk perception, regular HIV testing, and PrEP awareness. Educational and awareness programs that address the unique needs and barriers faced by these populations may enhance the general knowledge of PrEP, tackle negative perceptions, and improve the willingness to use PrEP to reduce HIV transmission rates.

## Figures and Tables

**Table 1:** Sociodemographic and HIV related Behavioral characteristics of the 497 articipants from key population communities in Kampala

Characteristic	Total (n %)	Fisherfolk (n%)	MSM (n%)	FSW (n %)	P-value
**Sociodemographic characteristics**					
**Age**					
18–39 (Young adult)	439 (88.3)	232 (82.0)	88 (94.6)	119 (98.3)	< 0.001
40–62 (Middle aged)	58 (11.7)	51 (18.0)	5 (5.4)	2 (1.7)	
**Location**					
Urban	359 (72.2)	271 (95.8)	76 (81.7)	12 (9.9)	< 0.001
Semi-urban	138 (27.8)	12 (4.2)	17 (18.3)	109 (90.1)	
**Education**					
None	46 (9.3)	39 (13.8)	2 (2.2)	5 (4.1)	< 0.001
Tertiary	72 (14.5)	10 (3.5)	51 (54.8)	11 (9.1)	
Primary	129 (26.0)	89 (31.5)	0	40 (33.1)	
Secondary	250 (50.3)	145 (51.2)	40 (43.0)	65 (53.7)	
**Employment**					
Not employed	170 (34.2)	49 (17.3)	50 (53.8)	71 (58.7)	0.500
Employed	327 (65.8)	234 (82.7)	43 (46.2)	50 (41.3)	
**Marital status**					
Not married	260 (52.31)	137 (48.4)	51 (54.8)	72 (59.5)	0.397
Married/Casual	237 (47.7)	146 (51.6)	42 (45.2)	49 (40.5)	
**HIV related Behavioral Characteristics**					
**HIV status awareness**					
No	104 (20.9)	76 (26.9)	21 (22.6)	7 (5.8)	0.745
Yes	393 (79.1)	207 (73.1)	72 (77.4)	114 (94.2)	
**HIV test in the past 6months**					
**Sociodemographic characteristics**					
**Age**					
No	269 (54.1)	181 (64.0)	58 (62.4)	30 (24.8)	0.001
Yes	228 (45.9)	102 (36.0)	35 (37.6)	91 (75.2)	
**Self reported HIV status**						< 0.001
Negative	318 (64.0)	188 (66.4)	58 (62.4)	72 (59.5)	
Positive	62 (12.5)	18 (6.4)	11 (11.8)	33 (27.3)	
Not-aware	117 (23.5)	77 (27.2)	24 (25.8)	16 (13.2)	
**Condom use**					
Always	133 (26.8)	50 (17.7)	46 (49.5)	37 (30.6)	0.001
Sometimes	237 (47.7)	125 (44.2)	32 (34.4)	80 (66.1)	
Never	127 (25.6)	108 (38.1)	15 (16.1)	4 (3.3)	
**High risk perception**					
No	93 (18.7)	36 (12.7)	23 (24.7)	34 (28.1)	< 0.001
Yes	404 (81.3)	247 (87.3)	70 (75.3)	87 (71.90)	
**Concerned about HIV**					
Not really	85 (17.1)	36 (12.7)	17 (18.3)	32 (26.4)	< 0.001
Yes	308 (62.0)	182 (64.3)	54 (58.1)	72 (59.5)	
Sometimes	104 (21.0)	65 (23.0)	22 (23.7)	17 (14.1)	
**PrEP awarness**						< 0.001
Yes	310 (62.4)	153 (54.1)	68 (73.1)	89 (73.6)	
No	187 (37.6)	130 (45.9)	25 (26.9)	32 (26.4)	

**Table 2:** Knowledge about different aspects of PrEP among the 497 participants from key population communities in Kampala

Question		Proportion
1. PrEP dose (How many times is someone supposed to take PrEP in a day for protection against HIV?)	Once a day	169 (34.0)
	Twice a day	20 (4.0)
	Three times a day	11 (2.2)
	Not sure	297 (59.8)
2. Duration on PrEP (How long can someone take PrEP?)	One month	10 (2.0)
	For as long as sexually I am active	23 (4.6)
	Not sure	464 (93.4)
3. I can still acquire HIV when am using PrEP		
	Yes	112 (22.5)
	No	77 (15.5)
	Not sure	308 (62.0)
4. PrEP reduces HIV infection by 90%	True	234 (47.1)
	False	70 (14.1)
	Not sure	193 (38.8)
5. I can still use other HIV preventive measure while am on PrEP		
	Yes	241 (48.5)
	No	75 (15.1)
	Not sure	181 (36.4)

**Table 3:** Bivariate analysis for factors associated with knowledge among the key populations

Characteristic	Fisherfolk		MSM		FSW	
	Coeff [95%CI]	P-value	Coeff [95%CI]	P-value	Coeff [95%CI]	P-value
**Sociodemographic characteristics**						
**Age**						
18–39 (Young adult)	1.00		1.00		1.00	
40–62 (Middle aged)	−0.18 [−0.52, 0.16]	0.308	0.23 [−0.81, 1.27]	0.664	−0.81 [−2.45, 0.83]	0.332
**Location**						
Urban	1.00		1.00		1.00	
Semi-urban	−0.88 [−1.57, −0.19]	0.012	0.31 [−0.28, 0.90]	0.303	−0.35 [−1.02, 0.33]	0.314
**Education**						
None	1.00		1.00		1.00	
Tertiary	0.05 [−0.73, 0.84]	0.894	0.09 [−1.43, 1.62]	0.905	1.48 [0.25, 2.71]	0.018
Primary	0.24 [−0.18, 0.67]	0.263	--	--	0.07 [−0.96, 1.11]	0.889
Secondary	0.51 [0.11, 0.92]	0.012	0.16 [−1.38, 1.69]	0.841	0.25 [−0.76, 1.26]	0.630
**Employment**						
Not employed	1.00		1.00		1.00	
Employed	−0.15 [−0.49, 0.20]	0.400	−0.58 [−1.04, −0.11]	0.015	0.18 [−0.23, 0.58]	0.391
**Marital status**						
Not married	1.00		1.00		1.00	
Married/casual	−0.03 [−0.29, 0.23]	0.807	0.12 [−0.33, 0.58]	0.592	0.76 [0.34, 1.18]	< 0.001
**HIV related Behavioral Characteristics**						
**HIV status awareness**						
No	1.00		1.00		1.00	
**Sociodemographic characteristics**						
Yes	0.48 [0.18, 0.78]	0.002	−0.04 [−0.58, 0.50]	0.891	0.73 [−0.17, 1.63]	0.110
**HIV test in the past 6 months**						
No	1.00		1.00		1.00	
Yes	−0.02 [−0.29, 0.25]	0.889	−0.19 [−0.66, 0.28]	0.426	0.60 [0.13, 1.07]	0.012
**Condom use**						
Always	1.00		1.00		1.00	
Sometimes	−0.16 [−0.53, 0.20]	0.384	−0.21 [−0.72, 0.29]		0.13 [−0.30, 0.57]	0.556
Never	−0.49 [−0.87, −0.11]	0.011	−0.74 [−1.40, −0.08]		0.13 [−1.02, 1.28]	0.824
**HIV risk perception**						
No	1.00		1.00		1.00	
Yes	0.49 [0.09–0.88]	0.017	0.73 [0.19–1.27]	0.008	0.21 [−0.24, 0.65]	0.362
**Concerned about HIV**						
Not really	1.00		1.00		1.00	
Yes	0.71 [0.30, 1.12]	0.001	0.99 [0.35, 1.62]	0.002	0.54 [0.07, 1.02]	0.025
Sometimes	0.53 [0.06, 1.01]	0.026	0.59 [−0.13, 1.31]	0.108	1.12 [0.43, 1.80]	0.001
**PrEP awareness**						
Yes	1.00		1.00		1.00	
No	−1.03 [−1.31, −1.05]	< 0.001	−1.81 [−2.44, −1.18]	< 0.001	−2.10 [−2.69, −1.51]	< 0.001

**Table 4:** Multivariate analysis for factors associated with knowledge in key populations

Characteristic	Unadjusted Coeff [95% CI]	Adjusted Coeff [95%]	P-value
**Fisherfolk key pipulation**			
**HIV concern**			
No	1.00	1.00	
Yes	0.71 [0.30, 1.12]	**0.54[0.11, 0.97]**	**0.013**
Sometimes	0.53 [0.06, 1.01]	0.38 [−0.10, 0.87]	0.117
**PrEP awareness**			
Yes	1.00	1.00	
No	−1.03 [−1.31, −1.05]	**−0.99 [−1.28, −0.70]**	**<0.001**
**FSW key population**			
**Education**			
No	1.00	1.00	
Yes	0.73 [0.19–1.27]	0.54 [−0.04, 1.12]	0.067
**PrEP awareness**			
Yes	1.00	1.00	
No	−1.81 [−2.44, −1.18]	**−1.74 [−2.38, −1.10]**	**<0.001**
**FSW key population**			
**Education**			
None	1.00	1.00	
Tertiary	1.48 [0.25, 2.71]	**1.53 [0.07, 2.99]**	**0.040**
Primary	0.07 [−0.96, 1.11]	0.17 [−1.09, 1.42]	0.797
Secondary	0.25 [−0.76, 1.26]	0.37 [−0.89, 1.62]	0.567
**Marital status**			
Not married	1.00	1.00	
Married/casual	0.76 [0.34, 1.18]	**0.57 [0.09, 1.05]**	**0.021**
**Concerned about HIV**			
Not really	1.00	1.00	
**Fisherfolk key population**			
**HIV concern**			
Yes	0.54 [0.07, 1.02]	**0.63 [0.07, 1.19]**	**0.027**
Sometimes	1.12 [0.43, 1.80]	**1.41 [0.61, 2.21]**	**0.001**
**PrEP awareness**r			
Yes	1.00	1.00	
No	−2.10 [−2.69, −1.51]	**−2.21 [−2.85, −1.57]**	**<0.001**

**Table 5:** Margin effects for factors associated with knowledge levels in key populations

Characteristic	Knowledge level	Coefficient [95% CI]	P-value
**Fisherfolk key population**			
**Concerned about HIV (Yes)**			
	No knowledge	**−0.16 [−0.30, −0.02]**	**0.022**
	Some knowledge	0.04 [−0.02, 0.11]	0.217
	Good knowledge	**0.12 [0.04, 0.20]**	**0.003**
**PrEP awareness (No)**			
	No knowledge	**0.28 [0.20, 0.37]**	**<0.001**
	Some knowledge	−0.04 [−0.10, 0.02]	0.159
	Good knowledge	**−0.24 [−0.31, −0.147]**	**<0.001**
**MSM key population**			
**HIV risk perception (Yes)**			
	No knowledge	−0.12 [−0.26, 0.02]	0.103
	Some knowledge	−0.03 [−0.07, 0.01]	0.177
	Good knowledge	**0.15 [0.01, 0.29]**	**0.044**
**PrEP awareness (No)**			
	No knowledge	**0.52 [0.32, 0.71]**	**<0.001**
	Some knowledge	−0.16 [−0.35, 0.02]	0.084
	Good knowledge	**−0.36 [−0.47, −0.25]**	**<0.001**
**FSW key population**			
**Education (Tertiary)**			
	No knowledge	−0.21 [−0.46, 0.03]	0.087
	Some knowledge	**−0.16 [−0.30, −0.03]**	**0.019**
	Good knowledge	**0.38 [0.08, 0.68]**	**0.014**
**Marital status (Married/casual)**			
	No knowledge	**−0.09 [−0.17, −0.01]**	**0.027**
	Some knowledge	−0.04 [−0.08, 0.01]	0.136
**Concerned about HIV (Yes)**			
	No knowledge	**−0.11 [−0.22, −0.01]**	**0.042**
	Some knowledge	**−0.01 [−0.06, 0.04]**	**0.834**
	Good knowledge	**0.12 [0.02, 0.22]**	**0.015**
**Concerned about HIV (Sometimes)**			
	No knowledge	**−0.22 [−0.35, −0.10]**	**0.001**
	Some knowledge	**−0.10 [−0.22, 0.02]**	**0.093**
	Good knowledge	**0.32 [0.14, 0.50]**	**<0.001**
**PrEP awareness (No)**			
	No knowledge	**0.61 [0.45, 0.76]**	**< 0.001**
	Some knowledge	**−0.33 [−0.48, −0.18]**	**< 0.001**
	Good knowledge	**−0.27 [−0.35, −0.19]**	**< 0.001**

## Data Availability

The datasets used and analyzed during this study are available from the corresponding author upon reasonable request because they involve other key population groups that are criminalized in Uganda.

## Abbreviations

HIV: human immunodeficiency virus
PrEP: Preexposure prophylaxis
SOMREC: School of Medicine Research and Ethics Committee
WHO: World Health Organization
